# Cigarette Smoke Extract Exposure Affects Innate Immune Response, Metabolic Rate, and Locomotor Activity of *Drosophila melanogaster*

**DOI:** 10.1007/s00408-026-00911-0

**Published:** 2026-07-09

**Authors:** Periklis Marnas, Lydia Giannakou, Eleanna Pitaraki, Elpiniki Stergiopoulou, Panagiotis Tzamalas, Athanasios Stefanos Giannopoulos, Maria Anastasia Parlantza, Konstantinos Gourgoulianis, Chrissi Hatzoglou, Erasmia Rouka, Stefan Lüpold, Sotirios G. Zarogiannis

**Affiliations:** 1 Department of Physiology, Faculty of Medicine, School of Health Sciences, University of Thessaly, BIOPOLIS, Larissa, Greece; 2 Department of Respiratory Medicine, Faculty of Medicine, School of Health Sciences, University of Thessaly, BIOPOLIS, Larissa, Greece; 3 Department of Nursing, School of Health Sciences, University of Thessaly, BIOPOLIS, Larissa, Greece; 4 Department of Evolutionary Biology and Environmental Studies, University of Zurich, Zurich, Switzerland

**Keywords:** Cigarette smoke extract, Innate immunity, Melanization, Oxidative stress, *Drosophila melanogaster*

## Abstract

**Purpose:**

Cigarette smoke (CS) disrupts innate immune homeostasis through oxidative stress and inflammatory signaling, contributing to respiratory diseases such as asthma and COPD. Because *Drosophila melanogaster* shares approximately 75% of disease-related human genes and possesses a well-characterized innate immune system without confounding adaptive responses, it represents a tractable model for investigating smoke-induced pathology.

**Methods:**

This study examined the effects of developmental cigarette smoke extract (CSE) exposure on innate immune activation, locomotor activity, and metabolic rate in *Drosophila melanogaster*.

**Results:**

Larvae exposed to 10–50% CSE exhibited dose-dependent increases in crystal-cell activation and melanized wound area, indicating heightened innate immune responsiveness likely driven by CSE-induced oxidative stress and hematopoietic dysregulation. Locomotor activity, assessed by negative geotaxis, was impaired at moderate concentrations in both sexes, with females showing greater sensitivity at lower doses, reflecting sex-specific differences. Metabolic rate measurements revealed a significant hypermetabolic response exclusively in male flies at 50% CSE, consistent with an elevated oxidative detoxification burden and vulnerability in males.

**Conclusion:**

Collectively, these findings demonstrate that CSE elicits a coordinated physiological stress response integrating immune activation with behavioral and metabolic dysfunction. This work supports *Drosophila melanogaster* as a translational model for dissecting the systemic consequences of smoke-induced innate immune dysregulation.

## Introduction

Cigarette smoke (CS) is a major threat to public health. It contains more than 4000 chemicals, including carbon monoxide, nicotine, cadmium, polycyclic aromatic hydrocarbons, which are harmful to human health [1, 2]. About 20% of the world’s population are smokers, while non-smokers also passively inhale CS and get exposed to its adverse effects [3, 4]. Smoking is a main risk factor for several respiratory diseases, including asthma and chronic obstructive pulmonary disease (COPD) [5, 6].

At the cellular level, CS acts as a potent inducer of oxidative stress and innate immune activation. Toxic components of CS lead to the production of reactive oxygen species (ROS), which induce damage to proteins, lipids and DNA [7, 8]. Beyond direct cytotoxicity, CS profoundly alters innate immune signaling, resulting in exaggerated inflammatory responses and impaired host defense. Indeed, smokers exhibit heightened inflammatory and immune responses following microbial stimulation, reflecting dysregulated innate immunity rather than effective immune protection [9, 10]. Smoke-induced oxidative stress and inflammation cause epithelial injury and cell death in the airway lining, which represents the first line of defense against inhaled pathogens and environmental insults.

Because innate immunity is evolutionarily conserved and centrally involved in smoke-induced pathology, tractable experimental models are essential for dissecting its systemic consequences [11, 12]. In addition to patient-based studies, animal models that allow mechanistic interrogation of immune, metabolic and behavioral outcomes are critical. *Drosophila melanogaster* is a powerful experimental model that shares approximately 75% of disease-related human genes and has been widely used to study conserved pathways involved in inflammation, oxidative stress, and metabolism [13, 14]. Importantly, the *Drosophila* tracheal system exhibits strong developmental and functional similarities to the mammalian respiratory system [15], rendering it suitable for modeling airway-related toxic exposures, including CS [14].

A unique advantage of *Drosophila* is the presence of a robust innate immune system in the absence of adaptive immunity, which allows direct assessment of innate immune mechanisms without confounding adaptive responses [16]. The *Drosophila* innate immune system relies on conserved cellular and humoral pathways, including hemocyte-mediated defenses and the melanization response [17, 18]. Melanization represents a rapid and energetically costly innate immune reaction, driven primarily by crystal cells, which release prophenoloxidases in response to tissue damage, infection, or environmental stress [19]. This response integrates immune activation with redox signaling and metabolic demand, making it a sensitive readout of systemic stress [20–23].

Innate immune activation is tightly coupled to organismal physiology. Immune responses impose significant energetic costs and interact with metabolic pathways, while oxidative stress and inflammation can impair neuromuscular function and behavior [24, 25]. Accordingly, behavioral traits such as locomotor activity and physiological traits such as metabolic rate serve as integrative phenotypes reflecting the organism-level impact of immune and oxidative stress. These traits are particularly relevant in the context of CS exposure, which is known to affect energy metabolism, mitochondrial function, and physical performance in higher organisms.

In this study, we used *D. melanogaster* as a model of cigarette smoke extract (CSE) exposure to investigate how a complex environmental toxicant influences innate immunity and whole-organism physiology. Specifically, we examined the effects of developmental CSE exposure on larval melanization and crystal-cell activation as indices of innate immune response, as well as on adult locomotor activity and metabolic rate as functional outcomes of systemic stress and rescued the phenotypes with N-acetyl-cysteine supplementation. By integrating immune, behavioral, and metabolic endpoints, this study aims to provide a coherent framework linking CS-induced innate immune activation to downstream physiological consequences.

## Materials and Methods

### Chemicals

Nipagin (methyl 4-hydroxybenzoate) (Sigma, H3647) was used as an antifungal in the growth medium. N-acetyl-cysteine (NAC) was used as an antioxidant agent in the growth medium in all experiments (UniPharma S.A., trebon N). Soda lime (Sigma, 72073) was used in the metabolic rate assay as an agent that adsorbs CO_2_ from the breathing gases.

### *Drosophila melanogaster* Strain and Culture

Wild-type *D. melanogaster* were collected using fruit traps outdoors in Zurich, Switzerland [13]. Male and female flies were separated under ice anesthesia post-eclosion. Flies were cultured on standard cornmeal–molasses–yeast–agar medium containing 7.5% w/v sucrose and 0.2% nipagin and maintained in 175 mL vials at 25 °C and 60% humidity under a 12 h:12 h light–dark cycle.

### Cigarette Smoke Extract preparation

A custom-made device was used to prepare CSE. The device consisted of an empty syringe, a syringe containing 5 mL of phosphate-buffered saline (PBS), a 3-way stopcock, and a pipette tip serving as a cigarette holder [13, 26]. Commercially available Marlboro^®^ cigarettes (10 mg tar, 0.8 mg nicotine, and 10 mg CO) were used. CS was first drawn through the 3-way stopcock into the empty syringe, then routed to the second syringe and bubbled through the PBS. Remaining smoke was discarded, and the procedure was repeated until the cigarette was consumed. The resulting PBS-CS mixture was combined with growth medium to prepare CSE at concentrations of 10%, 25%, and 50%.

### *Drosophila melanogaster* Cigarette Smoke Extract Exposure

Adult flies were allowed to mate on day 0 in vials containing medium with 10% PBS as a control or with 10%, 25%, and 50% CSE or supplemented with 1 mg/mL of NAC [27]. After three days, the adults were removed from the vials, and the progeny began to hatch on day 10. Both larval and adult medium contained CSE. Male and female flies were collected separately between days 10 and 12 and transferred to freshly prepared vials under all experimental conditions. Flies 2–4 days old were used for the experiments.

### Crystal Cells Counting Assay in Larvae

Immune activation was assessed by counting activated crystal cells, that are hemocytes responsible for initiating the melanization response [17]. For crystal cell visualization, twenty third-instar larvae were incubated in Eppendorf tubes at 70 °C for 10 min. After incubation, larvae were imaged under an optical microscope (ZEISS Axiostar Plus). For quantification, black puncta were counted as activated crystal cells. The experiment was repeated thrice.

### Melanization Assay in Larvae

The melanization assay, used to demonstrate the magnitude of the innate immune response, was performed as previously described [17]. Twenty third-instar larvae were wounded on the posterior side using a sterile needle (5 μm diameter). After 1 h, larvae were imaged under an optical stereoscope (OLYMPUS SZ30-ST), and the melanized area was quantified using Image J. The experiment was repeated thrice.

### Negative Geotaxis Assay in Adult Flies

Locomotor activity was assessed using the negative geotaxis assay, which measures the climbing behavior of the flies against gravity [28]. Thirty male or female flies from each experimental condition were divided into groups of six and transferred into five separate vials. Flies were gently tapped to the bottom of the vials, and a 10-cm mark was indicated on each vial. The percentage of flies that climbed above the 10-cm mark within 10 s was recorded. The experiment was repeated thrice.

### Metabolic Rate Assay in Adult Flies

Metabolic rate was measured by means of respirometry based on the CO_2_ production from the oxidative metabolism, as previously described [29]. Thirty flies from each experimental condition were divided into groups of six and transferred into five respirometers. CO_2_ production was recorded over 2 h following a 15-minute system equilibration. The experiment was repeated thrice.

### Statistical Analysis

The analyses were performed using GraphPad Prism (v 8.0) for Windows. Normality of the data was assessed with the D’ Agostino-Pearson test. The comparisons were performed using One-way or Brown-Forsythe and Welch ANOVA for parametric data or Kruskal-Wallis tests for non-parametric data. In the first case the Tukey or Dunnett T3 multiple comparisons post-hoc test was performed and in the latter the Dunn’s multiple comparisons post-hoc test was performed. Data were expressed as mean ± SEM. Values of *p <* 0.05 were considered as statistically significant.

## Results

### CSE Induces Innate Immune Activation in *Drosophila melanogaster* Larvae

**Fig. 1:**
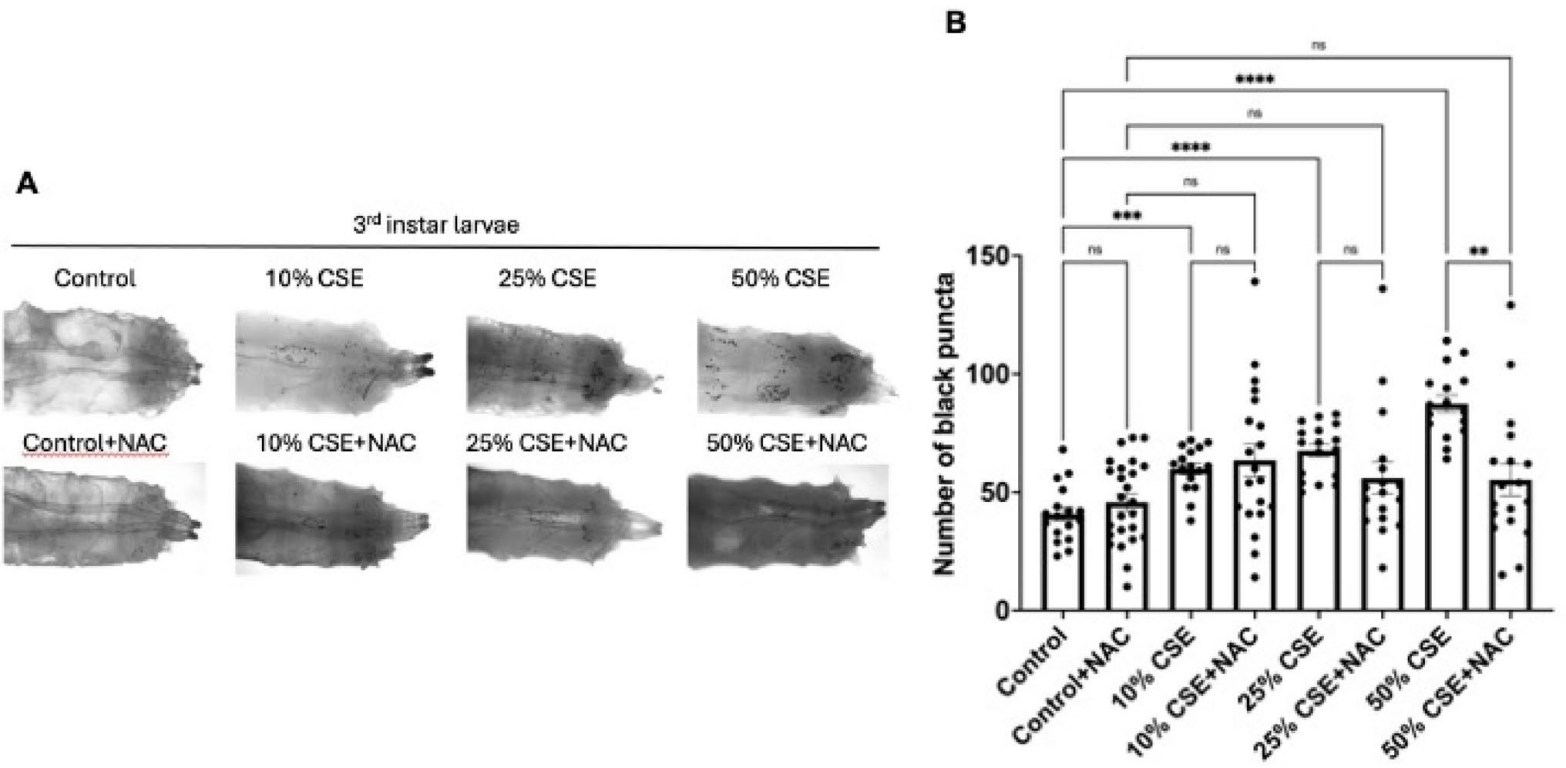
**A** Representative pictures of 3d instar larvae grown in 0% (control), 10%, 25%, and 50% CSE with or without NAC supplementation, that were place in 70 °C for 10 min to activate the crystal cells and migrate to the epidermis. **B** Number of black punctae (activated crystal cells) in the epidermis of 3d instar larvae grown in 0% (control), 10%, 25%, and 50% CSE with or without NAC supplementation. Values expressed in mean ± SEM, *N* = 3 experiments in each condition with *n* = 5–9 in each experiment; **p < 0.01, ****p < 0.0001 for comparisons with designated groups

Third-instar larvae were exposed to control, 10%, 25% or 50% conditions with or without NAC supplementation and the immune activation was determined by activating the melanization mechanism. Crystal cells, the main melanization activators, were counted as black puncta in the surface of the larvae (Fig. 1A**)**. Brown-Forsythe and Welch ANOVA with Dunnett post-hoc analysis was done. The results showed increasing numbers of larval crystal cells from control to 50% CSE conditions that were significantly increased as compared to Controls (Control: 40.76 ± 2.92, 10% CSE: 59.88 ± 2.42, 25% CSE: 67.59 ± 2.73, 50% CSE: 87.38 ± 3.62, *p* < 0.001; Fig. 1B). There was also significantly higher number of black puncta in the 50% group as compared to 10% and 25% groups (*p* < 0.001). Supplementation with NAC resulted in a significant reduction of the number of black puncta in the of 50% CSE + NAC as compared to 50% CSE without NAC supplementation (50% CSE + NAC: 55.28 ± 6.68; 50% CSE: 87.38 ± 3.62, *p* < 0.01; Fig. 1B).

**Fig. 2:**
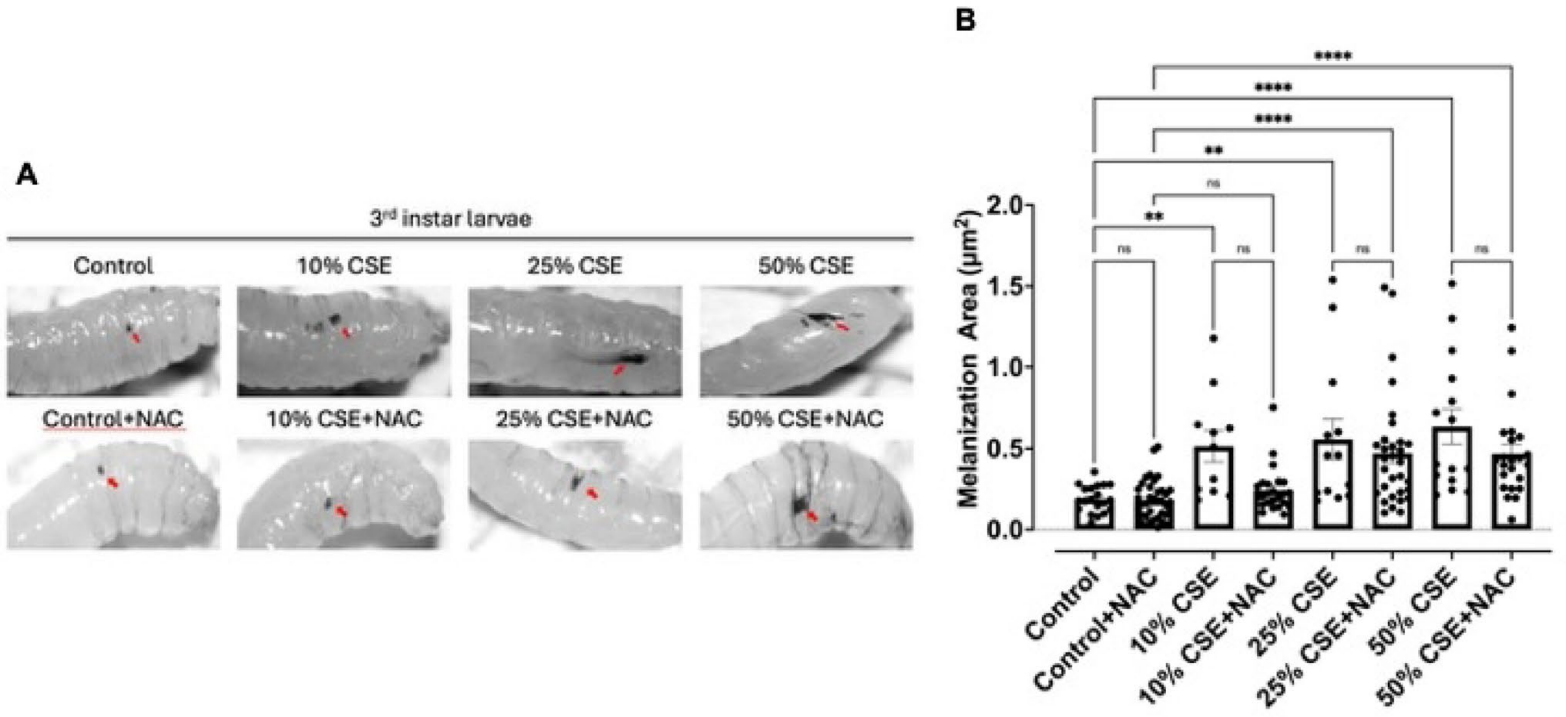
Effects of fine needle wound infliction on the melanization response after 0%, 10%, 25% and 50% CSE exposure of 3rd instar larvae, with or without NAC supplementation. Results are shown 1 h post wound infliction. **A** Representative pictures of 3d instar larvae 1 h post-wound with a fine needle grown in 0% (control), 10%, 25%, and 50% CSE, with or without NAC supplementation. **B** Analysis of melanized area of 3rd instar larvae 1-hour post-wound infliction measured in µm^2^ in the larvae that exhibited melanization response. Values expressed in mean ± SEM, *N* = 3 experiments in each condition with *n* = 4–12 in each experiment; **p < 0.01, ****p < 0.0001 for comparisons with designated groups

Subsequently, the surface of the melanized area of wounded larvae (Fig. 2A) was measured and quantified (Fig. 2B**).** One-way ANOVA for parametric data with Sidak post-hoc analysis was done. A significantly higher melanized area was observed in the larvae exposed to 10% CSE (Control: 0.19 ± 0.02, 10% CSE: 0.51 ± 0.10, *p =* 0.003), 25% CSE (Control: 0.19 ± 0.02, 25% CSE: 0.56 ± 0.13, *p =* 0.010) and 50% CSE (Control: 0.19 ± 0.02, 50% CSE: 0.64 ± 0.11, *p* < 0.0001). Supplementation with NAC resulted in a significant increase of the number of black puncta in the of 25% and 50% of CSE + NAC as compared to Controls + NAC supplementation (Control + NAC: 0.18 ± 0.02, 25% CSE + NAC: 0.47 ± 0.06, *p* < 0.0001) and 50% CSE (Control + NAC: 0.18 ± 0.02, 50% CSE + NAC: 0.47 ± 0.06, *p* < 0.0001) (Fig. 1B).

### CSE Exposure Reduces Adult *Drosophila melanogaster *Locomotor Activity in Low Concentrations

**Fig. 3:**
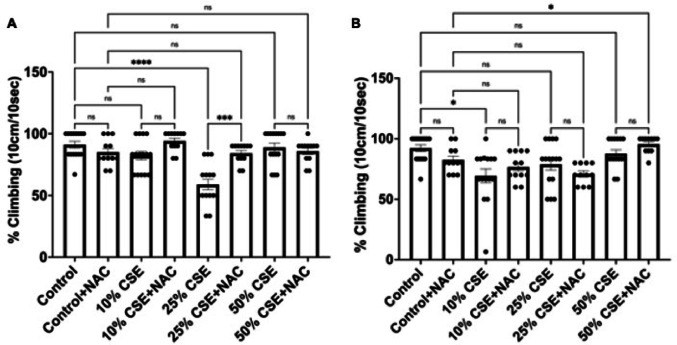
Effects of 10%, 25% and 50% CSE exposure, with or without NAC supplementation, on adult *D. melanogaster* negative geotaxis expressed as % of climbing 10 cm within 10 s. **A** Male *D. melanogaster* and **B** Female *D. melanogaster.* Values expressed in mean ± SEM, *N* = 3 experiments in each condition with *n* = 4–5 (10 flies in each one) in each experiment; *p < 0.05, ***p < 0.001, ****p < 0.0001 for comparisons with designated groups

Flies were exposed to 10%, 25%, and 50% CSE, and their locomotor activity was assessed. Male flies exposed to 25% CSE showed a significantly lower percentage of climbing compared to the control group (Control: 91.12 ± 2.74, 25% CSE: 58.86 ± 4.26; *p* < 0.001). There was no significant difference in the climbing activity among male flies exposed to 10% or 50% CSE compared to Controls (10% CSE: 82.19 ± 3.44, 50% CSE: 88.87 ± 3.52). Supplementation with NAC resulted in a significant increase in their climbing ability in the 25% CSE + NAC group as compared to 25% CSE (CSE: 58.86 ± 4.26, 25% CSE + NAC: 84.17 ± 2.29; *p* = 0.0003) (Fig. 3A).

Female flies exposed to 10% CSE showed a significantly lower percentage of climbing compared to the control group (Control: 92.21 ± 2.75, 10% CSE: 69.34 ± 5.71; *p* < 0.01), whereas those exposed to higher CSE concentrations did not (25% CSE: 78.87 ± 4.73, 50% CSE: 87.75 ± 3.03). In the NAC supplemented groups there was a significant increase in the mobility of the 50% CSE-NAC group as compared to Control-NAC group (Control-NAC:82.50 ± 3.05, 50% CSE-NAC: 95.45 ± 2.07; *p* < 0.05) (Fig. 3B).

### CSE Exposure Increases Adult Male *Drosophila melanogaster *Metabolic Rate in High Concentrations

**Fig. 4:**
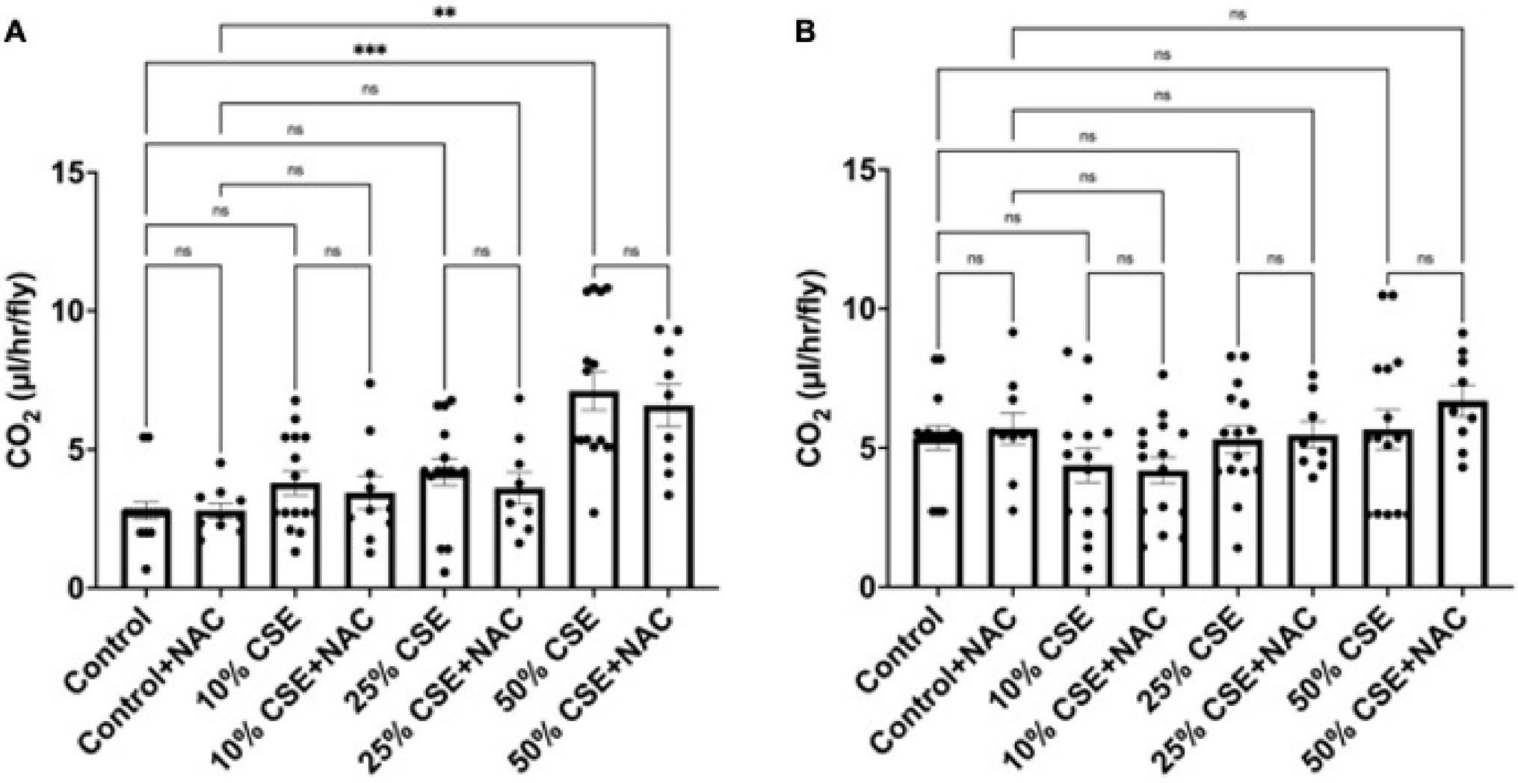
Effects of 10%, 25% and 50% CSE exposure, with or without NAC supplementation, on the metabolic rate of the flies measured as the production of CO_2_ (µl/hr/fly **A**) Male *D. melanogaster* and **B** Female *D. melanogaster.* Values expressed in mean ± SEM, *N* = 3 experiments in each condition with *n* = 3–5 (6 flies in each one) in each experiment; **p < 0.01, ***p < 0.001 for comparisons with designated groups

Flies exposed to varying CSE concentrations were further assessed for their metabolic rate. Male flies exposed to 50% CSE showed significantly higher CO_2_ production (µl/hr/fly) compared to Controls (Control: 2.80 ± 0.31, 50% CSE: 7.10 ± 0.68; *p* < 0.001), whilst lower concentrations resulted in no such effect (10% CSE: 3.79 ± 0.44, 25% CSE: 4.19 ± 0.48) (Fig. 4A).The same was the case with NAC supplementation comparing controls with 50% CSE-NAC (Control:-NAC 2.80 ± 0.26, 50% CSE-NAC: 6.60 ± 0.76; *p* < 0.01). By contrast, females showed no significant difference in CO_2_ production relative to Controls at any concentration without (Control: 5.37 ± 0.43, 10% CSE: 4.37 ± 0.60, 25% CSE: 5.31 ± 0.49, 50% CSE: 5.67 ± 0.71) or with NAC supplementation (Fig. 4B).

## Discussion

CS remains one of the most harmful environmental toxicants, exerting various effects on biological systems through its complex mixture of oxidants and reactive chemicals. A central mechanism underlying smoke-induced pathology is the disruption of innate immune homeostasis, driven by oxidative stress and chronic inflammatory signaling [10, 22]. Environmental pollutants, such as CS induce excessive ROS production, which leads not only to macromolecular damage but also to sustained activation of innate immune pathways and contributes to tissue injury, metabolic imbalance, and functional decline [22, 30].

Using *D. melanogaster* as an experimentally tractable model, our findings demonstrate that developmental exposure to CSE elicits a coordinated physiological stress response that integrates innate immune activation with behavioral and metabolic alterations. This integrative phenotype mirrors key features of smoke-induced pathology observed in higher organisms and highlights the utility of *D. melanogaster* for mechanistic studies of environmental lung toxicants [14, 21].

Exposure of larvae to CSE resulted in a pronounced increase in crystal-cell activation in a dose-dependent manner. Crystal cells are central effectors of the *D. melanogaster* innate immune response, and their expansion indicates heightened immune readiness rather than adaptive protection [11, 18]. This effect is likely mediated by CSE-induced oxidative stress, which is known to influence *D. melanogaster* hematopoiesis. Hematopoietic progenitors in the lymph gland are highly sensitive to redox stimuli, and increased ROS levels can promote their differentiation into mature immune effector cells, including crystal cells [20, 23]. The significant reduction in crystal cell activation by NAC at 50% CSE supports a causal role for oxidative stress in CSE-induced innate immune dysregulation.

Crystal-cell fate is tightly regulated by Notch signaling, which is itself modulated by oxidative and environmental stress. CSE-induced redox imbalance may bias progenitor differentiation toward the crystal-cell lineage or promote transdifferentiation of plasmatocytes into crystal cells, as has been observed under other stress conditions [31–33]. Functionally, expansion of the crystal-cell population primes the organism for melanization and wound repair, responses that may partially compensate for epithelial and metabolic damage elicited by smoke exposure.

Consistent with the observed increase in crystal-cell activation, CSE exposure of *D. melanogaster* also led to a significant expansion of the melanized area. Melanization represents a rapid, energetically costly innate immune response that integrates immune activation with oxidative chemistry [19]. Crystal cells serve as the primary reservoir of prophenoloxidases (PPO1 and PPO2), which are released upon activation or rupture to drive melanization, clot formation, and encapsulation responses [18, 34, 35]. Oxidative stress is also a potent inducer of melanization because ROS and damage cues stimulate phenoloxidase activity and promote melanin synthesis providing a direct biochemical pathway through which CSE induced redox imbalance would increase melanization output [19, 36]. Therefore, the enlarged melanized area observed in CSE-exposed larvae would likely reflect both an increased pool of melanizing cells and a heightened enzymatic activation within the melanization cascade. Moreover, studies on hematopoietic stress have shown that the increased differentiation of crystal cells under elevated ROS levels was accompanied by stronger melanization responses to immune or environmental challenges [23]. Provided that CSE disrupts epithelial integrity, redox homeostasis and immune signaling the enlarged melanized area would likely reflect a larger pool of melanizing crystal cells with heightened activation of the PPOs system. The melanization data revealed a dose-dependent partitioning of the immune response into an oxidatively driven component, amenable to NAC suppression at lower doses, and a residual component at higher doses that is antioxidant-resistant. This finding suggests that antioxidant strategies may be most effective in attenuating smoke-induced innate immune effector responses at early or low-level exposures but are unlikely to fully suppress melanization and thus inflammatory amplification under conditions of heavy or chronic smoke exposure. This response supports the notion that the increased melanized area due to CSE exposure reflects a systemic danger response in *D. melanogaster*.

Beyond immune activation, CSE exposure produced measurable functional consequences at the organismal level. Locomotor activity in adult flies, assessed by negative geotaxis, was reduced at moderate CSE concentrations in both males and females (25% and 10% respectively). Negative geotaxis is a sensitive integrative measure of neuromuscular integrity, metabolic capacity, and stress tolerance, and impairments in climbing behavior have been consistently linked to oxidative stress and toxicant exposure in *D. melanogaster* [37, 38]. The non-linear dose–response pattern observed here suggests that moderate CSE exposure may be sufficient to disrupt neuromuscular coordination, while higher concentrations may activate compensatory stress-response pathways that transiently preserve locomotor function [21]. NAC effectively rescued the dose at which locomotor impairment was most clearly oxidatively driven (25% CSE in males), while revealing sex-specific hormetic responses at higher doses that would not be apparent without antioxidant co-treatment.

Sex-specific differences in locomotor impairment likely reflect intrinsic differences in oxidative stress resilience. Female *D. melanogaster* have been shown to exhibit higher antioxidant enzyme activity and lower ROS accumulation under stress compared to males, which may shift the threshold at which locomotor deficits become apparent [39]. These findings parallel sex-specific differences observed in smoke-induced functional decline in mammals and emphasize the importance of considering sex as a biological variable in toxicological models [40].

Metabolic measurements in adult flies further revealed a sex-dependent physiological response to CSE exposure. Only male flies showed a significant increase in the metabolic rate at the highest CSE concentration (50%), indicating a sex-specific hypermetabolic stress response. Male *D. melanogaster* typically exhibit lower antioxidant reserves than females [39, 41]. High-dose CSE is expected to elevate ROS and likely imposes a substantial detoxification burden, which increases energetic demand and oxygen consumption [24]. This hypermetabolic state may represent an adaptive but energetically costly attempt to maintain homeostasis under extreme oxidative challenge. However, the failure of NAC to normalize male metabolic rate at 50% CSE is suggestive of the fact that the hypermetabolic response in this exposure range probably involves mechanisms multimodal pharmacological approaches targeting perhaps mitochondrial integrity and bioenergetics rather than upstream ROS scavenging alone.

The metabolic rates observed in this study were significantly lower than those reported in our previous work, which may reflect the significance of CSE exposure from the beginning of life for the flies (from the egg stage) until adulthood, as opposed to only adult stage exposure previously [14]. This confirms the strong correlation of metabolic profiles with actual developmental stages throughout the *D. melanogaster* life cycle, suggesting the presence of age-dependent metabolic gradients in males that are less pronounced in females, which further contributes to sex-specific vulnerability [42]. Mitochondrial function in males appears particularly sensitive to oxidative stress, providing a plausible mechanistic explanation for the male-restricted metabolic phenotype observed under high CSE exposure [40, 43].

The apparent divergence across our three endpoints reflects CSE effects intersecting in the dual function of crystal cells as both immune effectors and oxygen carriers. It has been recently demonstrated that crystal cells control internal oxygen homeostasis via PPO2 phase transitions and that their loss produces systemic hypoxia under normoxic conditions [44]. CSE induction of crystal cells resulting in melanization sets a functional trade-off between immune execution and oxygen transport. The resulting internal hypoxia activates the HIF-1α homolog Sima, which modifies neuromuscular junction architecture via glial Wnt/Wingless signaling and impairs coordinated locomotion [45], while mitochondrial ROS produced under oxidative challenge feed into this same HIF-1α axis to alter neuromuscular plasticity and behavior [46]. These mechanisms explain the locomotor impairment at moderate CSE concentrations. At high CSE doses, HIF-1α-dependent compensatory pathways stabilize synaptic transmission at an increased energetic cost, manifesting as the hypermetabolic CO_2_ elevation observed exclusively in males, a pattern consistent with the established principle that *Drosophila* immune activation diverts dietary carbon toward hematopoietic energy demands via inter-organ adenosine signaling [47], and that insect muscle is directly co-opted during immune responses at the cost of reduced motor output [48]. *Drosophila* integrates CO_2_ and oxygen chemosensory inputs through a brain-fat body signaling axis to regulate immune cell differentiation [49]. The elevated CO_2_ production we measure may itself partially be fed back into immune regulation, coupling the metabolic and immune readouts. Males could be more susceptible to the immune-respiratory trade-off generated by crystal cell activation, explaining why only males exhibit the hypermetabolic response at 50% CSE. This would require further experimentation to be verified. Females, whose systemic immune capacity and antioxidant buffering are relatively higher [50], shift the threshold at which locomotor deficits emerge to lower CSE concentrations while avoiding the male-type metabolic escalation entirely. These non-parallel dose response profiles across endpoints seem therefore as a predictable consequence of the sex-dependent balance between immune oxygen cost, HIF-1α-mediated neuromuscular compensation and metabolic reserve capacity.

Taken together, our findings reveal that CSE induces a coordinated physiological stress response in *D. melanogaster*, characterized by innate immune activation, increased melanization, sex-specific locomotor impairment, and differential metabolic activation. By linking immune activation to locomotor and metabolic outcomes, this study reinforces the concept that innate immunity operates as a central hub connecting environmental stress to whole-organism physiology [12]. These results support the use of *D. melanogaster* as a translational model for studying smoke-induced immune and metabolic dysfunction and provide a framework for future investigations into the molecular pathways linking oxidative stress, immunity, and functional decline.

## Data Availability

All data supporting the findings of this study are available within the paper .

## References

[1] Zuo H (2020) Cigarette smoke exposure alters phosphodiesterases in human structural lung cells. Am J Physiol Lung Cell Mol Physiol 318(1):L59–L64. 10.1152/ajplung.00319.2019 31664853 10.1152/ajplung.00319.2019

[2] Nicolaou L, Checkley W (2021) Differences between cigarette smoking and biomass smoke exposure: An in silico comparative assessment of particulate deposition in the lungs. Environ Res 197:111116. 10.1016/j.envres.2021.111116 33823195 10.1016/j.envres.2021.111116 PMC8187290

[3] Soleimani F, Dobaradaran S, De-la-Torre GE, Schmidt TC, Saeedi R (2022) Content of toxic components of cigarette, cigarette smoke vs cigarette butts: a comprehensive systematic review. Sci Total Environ 813:152667. 10.1016/j.scitotenv.2021.152667 34963586 10.1016/j.scitotenv.2021.152667

[4] McAdam K et al (2016) Influence of cigarette circumference on smoke chemistry, biological activity, and smoking behaviour. Regul Toxicol Pharmacol 82:111–126. 10.1016/j.yrtph.2016.09.010 27634061 10.1016/j.yrtph.2016.09.010

[5] Hajat C, Stein E, Ramstrom L, Shantikumar S, Polosa R (2021) The health impact of smokeless tobacco products: a systematic review. Harm Reduct J 18(1):123. 10.1186/s12954-021-00557-6 34863207 10.1186/s12954-021-00557-6 PMC8643012

[6] Christenson SA, Smith BM, Bafadhel M, Putcha N (2022) Chronic obstructive pulmonary disease. Lancet 399(10342):2227–2242. 10.1016/S0140-6736(22)00470-6 35533707 10.1016/S0140-6736(22)00470-6

[7] Agraval H, Chu HW (2022) Lung organoids in smoking research: current advances and future promises. Biomolecules 12(10):1463. 10.3390/biom12101463 36291672 10.3390/biom12101463 PMC9599326

[8] Caliri AW, Tommasi S, Besaratinia A (2021) Relationships among smoking, oxidative stress, inflammation, macromolecular damage, and cancer. Mutat Res Rev Mutat Res 787:108365. 10.1016/j.mrrev.2021.108365 34083039 10.1016/j.mrrev.2021.108365 PMC8287787

[9] Saint-André V et al (2024) Smoking changes adaptive immunity with persistent effects. Nature 626(8000):827–835. 10.1038/s41586-023-06968-8 38355791 10.1038/s41586-023-06968-8 PMC10881394

[10] Aghapour M, Raee P, Moghaddam SJ, Hiemstra PS, Heijink IH (2018) Airway epithelial barrier dysfunction in chronic obstructive pulmonary disease: role of cigarette smoke exposure. Am J Respir Cell Mol Biol 58(2):157–169. 10.1165/rcmb.2017-0200TR 28933915 10.1165/rcmb.2017-0200TR

[11] Lemaitre B, Hoffmann J (2007) The host defense of Drosophila melanogaster. Annu Rev Immunol 25:697–743. 10.1146/annurev.immunol.25.022106.141615 17201680 10.1146/annurev.immunol.25.022106.141615

[12] Buchon N, Silverman N, Cherry S (2014) Immunity in Drosophila melanogaster: from microbial recognition to whole- organism physiology. Nat Rev Immunol 14(12):796–810. 10.1038/nri3763 25421701 10.1038/nri3763 PMC6190593

[13] Giannopoulos A-S et al (2023) The effect of cigarette smoke extract exposure on the size and sexual behaviour of Drosophila melanogaster. Environ Toxicol Pharmacol 104:104325. 10.1016/j.etap.2023.104325 37995887 10.1016/j.etap.2023.104325

[14] Marnas P et al (2025) Modeling COPD in Drosophila melanogaster by cigarette smoke inhalation: functional changes and alterations in the expression of COPD-relevant orthologous genes. Am J Physiol Regul Integr Comp Physiol 329(1):R13–R19. 10.1152/ajpregu.00056.2025 40392629 10.1152/ajpregu.00056.2025

[15] Scholl A, Ndoja I, Jiang L (2021) Drosophila trachea as a novel model of COPD. Int J Mol Sci 22(23):12730. 10.3390/ijms222312730 34884534 10.3390/ijms222312730 PMC8658011

[16] Bretscher AJ et al (2015) The Nimrod transmembrane receptor Eater is required for hemocyte attachment to the sessile compartment in Drosophila melanogaster. Biol Open 4(3):355–363. 10.1242/bio.201410595 25681394 10.1242/bio.201410595 PMC4359741

[17] Dudzic JP, Kondo S, Ueda R, Bergman CM, Lemaitre B (2015) Drosophila innate immunity: regional and functional specialization of prophenoloxidases. BMC Biol 13:81. 10.1186/s12915-015-0193-6 26437768 10.1186/s12915-015-0193-6 PMC4595066

[18] Vlisidou I, Wood W (2015) Drosophila blood cells and their role in immune responses. FEBS J 282(8):1368–1382. 10.1111/febs.13235 25688716 10.1111/febs.13235

[19] Cerenius L, Söderhäll K (2021) Immune properties of invertebrate phenoloxidases. Dev Comp Immunol 122:104098. 10.1016/j.dci.2021.104098 33857469 10.1016/j.dci.2021.104098

[20] Owusu-Ansah E, Banerjee U (2009) Reactive oxygen species prime Drosophila haematopoietic progenitors for differentiation. Nature 461(7263):537–541. 10.1038/nature08313 19727075 10.1038/nature08313 PMC4380287

[21] Prange R et al (2018) A Drosophila model of cigarette smoke induced COPD identifies Nrf2 signaling as an expedient target for intervention. Aging 10(8):2122–2135. 10.18632/aging.101536 30153653 10.18632/aging.101536 PMC6128429

[22] Lugg ST, Scott A, Parekh D, Naidu B, Thickett DR (2022) Cigarette smoke exposure and alveolar macrophages: mechanisms for lung disease. Thorax 77(1):94–101. 10.1136/thoraxjnl-2020-216296 33986144 10.1136/thoraxjnl-2020-216296 PMC8685655

[23] Sinenko SA, Shim J, Banerjee U (2011) Oxidative stress in the haematopoietic niche regulates the cellular immune response in Drosophila. EMBO Rep 13(1):83–89. 10.1038/embor.2011.223 22134547 10.1038/embor.2011.223 PMC3246251

[24] Musselman LP et al (2011) A high-sugar diet produces obesity and insulin resistance in wild-type Drosophila. Dis Model Mech 4(6):842–849. 10.1242/dmm.007948 21719444 10.1242/dmm.007948 PMC3209653

[25] Lochmiller RL, Deerenberg C (2000) Trade-offs in evolutionary immunology: just what is the cost of immunity? Oikos 88(1):87–98. 10.1034/j.1600-0706.2000.880110.x

[26] Jagirdar RM et al (2024) Short term exposure of sheep tracheal epithelium to cigarette smoke extract reduces ENaC current: a pilot study. In Vivo 38(5):2294–2299. 10.21873/invivo.13694 10.21873/invivo.13694 PMC11363775 39187341

[27] Niraula P, Kim MS (2019) N-Acetylcysteine extends lifespan of Drosophila via modulating ROS scavenger gene expression. Biogerontology 20(4):533–543. 10.1007/s10522-019-09815-4 31115735 10.1007/s10522-019-09815-4

[28] El-Merhie N et al (2021) Sex dependent effect of maternal e-nicotine on F1 Drosophila development and airways. Sci Rep 11(1):4441. 10.1038/s41598-021-81607-8 33627715 10.1038/s41598-021-81607-8 PMC7904947

[29] Yatsenko AS, Marrone AK, Kucherenko MM, Shcherbata HR (2014) Measurement of metabolic rate in Drosophila using respirometry. J Vis Exp no 88:e51681. 10.3791/51681 10.3791/51681 PMC4205100 24998593

[30] Celebi Sözener Z, Cevhertas L, Nadeau K, Akdis M, Akdis CA (2020) Environmental factors in epithelial barrier dysfunction. J Allergy Clin Immunol 145(6):1517–1528. 10.1016/j.jaci.2020.04.024 32507229 10.1016/j.jaci.2020.04.024

[31] Duvic B, Hoffmann JA, Meister M, Royet J (2002) Notch signaling controls lineage specification during Drosophila larval hematopoiesis. Curr Biol 12(22):1923–1927. 10.1016/s0960-9822(02)01297-6 12445385 10.1016/s0960-9822(02)01297-6

[32] Letourneau M, Lapraz F, Sharma A, Vanzo N, Waltzer L, Crozatier M (2016) Drosophila hematopoiesis under normal conditions and in response to immune stress. FEBS Lett 590(22):4034–4051. 10.1002/1873-3468.12327 27455465 10.1002/1873-3468.12327

[33] Katz MJ et al (2025) Autophagy controls differentiation of Drosophila blood cells by regulating Notch levels in response to nutrient availability. Nat Commun 16(1):5858. 10.1038/s41467-025-58389-y 40595449 10.1038/s41467-025-58389-y PMC12215389

[34] De Gregorio E, Spellman PT, Rubin GM, Lemaitre B (2001) Genome-wide analysis of the Drosophila immune response by using oligonucleotide microarrays. Proc Natl Acad Sci USA 98(22):12590–12595. 10.1073/pnas.221458698 11606746 10.1073/pnas.221458698 PMC60098

[35] Bidla G, Dushay MS, Theopold U (2007) Crystal cell rupture after injury in Drosophila requires the JNK pathway, small GTPases and the TNF homolog Eiger. J Cell Sci 120:1209–1215. 10.1242/jcs.03420 17356067 10.1242/jcs.03420

[36] Mucha M, Skrzydlewska E, Gęgotek A (2025) Natural protection against oxidative stress in human skin melanocytes. Commun Biol 8(1):1283. 10.1038/s42003-025-08725-1 40858814 10.1038/s42003-025-08725-1 PMC12381135

[37] Linderman JA, Chambers MC, Gupta AS, Schneider DS (2012) Infection-related declines in chill coma recovery and negative geotaxis in Drosophila melanogaster. PLoS ONE 7(9):e41907. 10.1371/journal.pone.0041907 23028430 10.1371/journal.pone.0041907 PMC3441536

[38] Olalekan Abolaji A et al (2014) Involvement of oxidative stress in 4-vinylcyclohexene-induced toxicity in Drosophila melanogaster. Free Radic Biol Med 71:99–108. 10.1016/j.freeradbiomed.2014.03.014 24681254 10.1016/j.freeradbiomed.2014.03.014

[39] Niveditha S, Deepashree S, Ramesh SR, Shivanandappa T (2017) Sex differences in oxidative stress resistance in relation to longevity in Drosophila melanogaster. J Comp Physiol B 187(7):899–909. 10.1007/s00360-017-1061-1 28261744 10.1007/s00360-017-1061-1

[40] Tower J (2006) Sex-specific regulation of aging and apoptosis. Mech Ageing Dev 127(9):705–718. 10.1016/j.mad.2006.05.001 16764907 10.1016/j.mad.2006.05.001

[41] Magwere T, Chapman T, Partridge L (2004) Sex differences in the effect of dietary restriction on life span and mortality rates in female and male Drosophila melanogaster. J Gerontol Biol Sci Med Sci 59(1):3–9. 10.1093/gerona/59.1.b3 10.1093/gerona/59.1.b3 14718480

[42] An PNT, Yamaguchi M, Fukusaki E (2017) Metabolic profiling of Drosophila melanogaster metamorphosis: a new insight into the central metabolic pathways. Metabolomics 13(3):29. 10.1007/s11306-017-1167-1

[43] Lushchak VI (2011) Adaptive response to oxidative stress: bacteria, fungi, plants and animals. Comp Biochem Physiol C Toxicol Pharmacol 153(2):175–190. 10.1016/j.cbpc.2010.10.004 20959147 10.1016/j.cbpc.2010.10.004

[44] Shin M et al (2024) Drosophila immune cells transport oxygen through PPO2 protein phase transition. Nature 631(8020):350–359. 10.1038/s41586-024-07583-x 38926577 10.1038/s41586-024-07583-x PMC11236712

[45] Chen P-Y, Tsai Y-W, Cheng Y-J, Giangrande A, Chien C-T (2019) Glial response to hypoxia in mutants of NPAS1/3 homolog Trachealess through Wg signaling to modulate synaptic bouton organization. PLoS Genet 15(8):e1007980. 10.1371/journal.pgen.1007980 31381576 10.1371/journal.pgen.1007980 PMC6695205

[46] Sobrido-Cameán D et al (2025) Mitochondrial ROS and HIF-1α signaling mediate synaptic plasticity in the critical period. PLOS Biol 23(8):e3003338. 10.1371/journal.pbio.3003338 40802851 10.1371/journal.pbio.3003338 PMC12367176

[47] Lazzaro BP (2015) Adenosine Signaling and the Energetic Costs of Induced Immunity. PLOS Biol 13(4):e1002136. 10.1371/journal.pbio.1002136 25915419 10.1371/journal.pbio.1002136 PMC4411026

[48] Adamo SA et al (2023) Muscle in the caterpillar Manduca sexta responds to an immune challenge, but at a cost, suggesting a physiological trade-off. J Exp Biol 226(14):jeb245861. 10.1242/jeb.245861 37334669 10.1242/jeb.245861 PMC10399994

[49] Cho B, Spratford CM, Yoon S, Cha N, Banerjee U, Shim J (2018) Systemic control of immune cell development by integrated carbon dioxide and hypoxia chemosensation in Drosophila. Nat Commun 9(1):2679. 10.1038/s41467-018-04990-3 29992947 10.1038/s41467-018-04990-3 PMC6041325

[50] Kubiak M, Tinsley MC (2017) Sex-specific routes to immune senescence In Drosophila melanogaster. Sci Rep 7(1):10417. 10.1038/s41598-017-11021-6 28874758 10.1038/s41598-017-11021-6 PMC5585412

